# Sensory exotropia versus sensory esotropia: A comparative clinical features study

**DOI:** 10.1016/j.optom.2024.100516

**Published:** 2024-04-24

**Authors:** Mohammad Reza Akbari, Alaa Alghurab, Masoud Khorrami-Nejad, Elham Azizi, Babak Masoomian

**Affiliations:** a Translational Ophthalmology Research Center, Farabi Eye Hospital, Tehran University of Medical Sciences, Tehran, Iran; b Optometry Department, School of Rehabilitation, Tehran University of Medical Sciences, Tehran, Iran; c Mashhad University of Medical Sciences, Mashhad, Iran

**Keywords:** Sensory esotropia, Sensory exotropia, Amblyopia, Refractive error

## Abstract

**Purpose:**

This study aimed to compare the preoperative clinical features of patients with sensory esotropia (ET) and sensory exotropia (XT).

**Methods:**

In a retrospective study, the medical records of 13,252 patients who underwent strabismus surgery were reviewed at the Farabi Eye Hospital, Iran, from 2012 to March 2022. There were 1017 patients with sensory horizontal strabismus whose, in their worse eye, had corrected distance visual acuity (CDVA) equal to or <20/160 tested with the Snellen chart.

**Results:**

The mean age of patients was 29.0 ± 12.4 years [574 (56.4%) males and 443 (43.6%) females]. Sensory XT and ET were observed in 717 (70.5%) and 300 (29.5%) patients, respectively (*P*<.001). The mean CDVA in the strabismic and non-strabismic eyes was 1.40 ± 0.75 and 0.05 ± 0.13, respectively (*P*<.001). Also, the CDVA in the strabismic eyes was significantly worse in the patients with sensory XT than in the patients with sensory ET (*P*<.001). Sphere and spherical equivalent (SE) components were more hyperopic in both eyes of patients with sensory ET than sensory XT (*P*<.001). In sensory ET group, the mean horizontal deviation at far and near was significantly higher than the sensory XT group (both *P*<.001). The prevalence of moderate and severe amblyopia among all patients with sensory strabismus was 274 (26.9%) and 727 (71.5%), respectively (*P*<.001). There were 398 (39.1%) patients who needed more than one surgery.

**Conclusion:**

The frequency of sensory XT was about 2.5 times more than the sensory ET. Most patients with sensory ET were operated at a younger age, had better CDVA, more hyperopic spherical and SE, and higher angle of deviation compared with patients with sensory XT. The chance of reoperation in patients with sensory strabismus was about 40%.

## Introduction

Sensory fusion and ocular motor are two essential mechanisms that support ocular alignment. If these two mechanisms are compromised via sensory or motor fusion deficits, it can lead to difficulties during visual tasks.^1^^,^^2^ When there is a loss of visual acuity in one eye, sensory fusion is impaired, and strabismus may occur due to the primary sensory impairment. Sensory strabismus refers to a secondary ocular deviation due to the disruption of sensory fusion that occurs mainly after loss or severe reduction in visual acuity in one or both eyes.^3^, ^4^, ^5^ Numerous risk factors can induce sensory strabismus. From the clinical perspective, every persistent unilateral or bilateral vision loss at any age can contribute to developing secondary or sensory strabismus. For example, anisometropia, cataracts, corneal opacities, and optic nerve anomalies are the most frequent causes of poor vision and resultant sensory strabismus.

Sensory strabismus may be horizontal or vertical or a combination of both^3^; however, most reports indicate the development of horizontal deviation as sensory strabismus.^3^^,^^6^, ^7^, ^8^, ^9^, ^10^ Most studies on the prevalence of sensory strabismus reported a higher prevalence for sensory exotropia than sensory esotropia.^6^^,^^10^, ^11^, ^12^ Several factors can determine the direction of sensory strabismus, namely, age at the time of visual impairment, refractive error in the normal eye, degree of visual acuity, and anatomical factors^3^^,^^4^^,^^13^^,^^14^; however, there is still no consensus. Sidikaro and von Noorden^5^ and Min et al.^8^ found that esotropia is relatively dominant when visual impairment occurs immediately after birth or before the age of 5 years. Havertape et al.^3^ reported that esotropia was dominant for congenital visual loss occurring within six months of age, and sensory exotropia for acquired visual loss over 22 months of age is the predominant form of sensory strabismus.

There might be several reasons that whether a sensory esotropia develops or a sensory exotropia. So far, most studies have focused on the underlying reasons for developing sensory strabismus, or they have demonstrated that the direction of sensory strabismus was significantly associated with the age at the onset of visual impairment and the refractive error in the sound eye^7^^,^^15^^,^^16^; however, they did not report on other clinical differences including visual acuity, the magnitude of deviation and age at the time of surgery between the sensory esotropia and sensory exotropia. In this retrospective study, we compared the clinical features of a large sample of patients (1017) with sensory esotropia and exotropia who underwent strabismus surgery. This would help clinicians become more familiar with various clinical features in patients with sensory esotropia and sensory exotropia.

### Patients and methods

The medical records of 13,252 patients who underwent strabismus surgery were reviewed retrospectively at Farabi Eye Hospital, Iran, from 2012 to March 2022. Of these, 1017 patients included in the analysis and met the inclusion criteria for sensory strabismus. The study was conducted after approval of the study protocol by the Institutional Review Board of Tehran University of Medical Sciences and was performed according to the tenets of the Declaration of Helsinki. Sensory strabismus was defined as a horizontal or vertical deviation, evaluated with the modified Krimsky test and secondary to diminished vision in one or both eyes with corrected distance visual acuity equal to or worse than 2/10 (0.7 logMAR) in the worse eye.^10^^,^^17^^,^^18^ The inclusion and exclusion criteria were determined as follows:

Inclusion criteria:

▪ Corrected distance visual acuity in the worse eye equal or worse than 2/10 (0.7 logMAR), tested with the Snellen visual acuity chart (in patients older than 3.5 years old with enough cooperation for conducting the test).
▪ Presence of sensory esotropia or exotropia evaluated with the modified Krimsky test method.
▪ The main causes for developing sensory strabismus included anisometropia, congenital cataract, corneal opacities, congenital glaucoma, perforating injury, retinal detachment, optic nerve anomalies, and Persistent Hyperplastic Primary Vitreous (PHPV).

Exclusion criteria:

▪ Strabismus without any underlying organic cause or severe anisometropia.
▪ Systemic disabilities such as motor or mental disabilities, plagiocephalic syndromes, craniofacial anomalies.
▪ History of previous ocular surgery, including strabismus or refractive surgery.

Then, the following pre-surgery data were collected and analyzed: spherical, cylindrical, and spherical equivalent refractive error, best-corrected distance visual acuity, angle of deviation at distance and near (prism diopter), age at the time of surgery and the severity of amblyopia.

Myopia was defined as a spherical equivalent of −0.50 diopter (D) or more. Hyperopia was defined as a spherical equivalent of +0.50 D or greater.^19^^,^^20^ Spherical equivalent refractive error was calculated by adding half of the astigmatism power in the minus cylinder to the spherical refractive error component.

Although this study had a retrospective design, the routine ophthalmic examinations in our academic eye center included: refractive error measurement using an autorefraction (Topcon KR-8900, Topcon Corporation, Tokyo, Japan), and then the confirmation of the results by the Heine beta 200 retinoscope (Heine Optotechnik, Herrsching, Germany), visual acuity measurement using the Snellen visual acuity chart at 6 m, and evaluating and measuring the angle of deviation using the modified Krimsky test (if the ocular fixation was poor) and recording the results in prism diopter (Δ).^18^ In this test, a decentered corneal reflex in the strabismic eye is gradually centered by increasing the prism power placed on the non-strabismic eye with the apex towards the direction of the deviation.^21^ Then, eye movements, as well as the presence of any overshoot and undershoot of the extraocular muscles, were tested by the motility test. Slit lamp evaluation and fundus examination were also performed for all patients. Throughout the examinations, refractive correction was worn by all participants. Only patients who had complete medical records were included. The records also needed to have a definite diagnosis of sensory strabismus and be eligible for strabismus surgery. Amblyopia was defined as an interocular difference of two lines or more in visual acuity or visual acuity equal or worse than 0.2 logMAR with the best optical correction in the presence of an amblyogenic factor.^22^ The amblyogenic factors were defined as the presence of an anisometropia (difference in myopia, hyperopia, and astigmatism equal or >3.00 D, 1.00 D, and 1.50 D, respectively), a constant unilateral heterotropia at distance and/or near fixation or combined anisometropia and strabismus. Amblyopic patients were divided into three severities: mild (best corrected visual acuity in the amblyopic eye of 0.2 logMAR), moderate (best corrected visual acuity in the amblyopic eye 0.3–0.7 logMAR), and severe (≥0.8 logMAR) in unilateral cases and best-corrected distance visual acuity equal or <0.3 logMAR in bilateral cases.^23^, ^24^, ^25^

For sensory strabismus surgery, the preferred approach was recession-resection surgery on the deviated eye.^26^ This involved recessions and resections on the extraocular muscles (most commonly the medial and lateral rectus muscles) to reduce the ocular misalignment. The specific extraocular muscles operated on depending on the direction of deviation. For example, lateral rectus recession and medial rectus resection were typically performed in sensory exotropia. The amount of recession and resection was determined based on the preoperative angle of deviation. In some cases, vertical extraocular muscles were also operated on to correct associated vertical deviations.

The data collected was analyzed using the SPSS-26 software (IBM Inc., Chicago, USA). Normal data distribution was tested by the Shapiro–Wilk test, and according to the normal distribution of the data, independent samples *t*-test was used to compare the mean values of variables between the sensory esotropia and sensory exotropia groups. Paired *t*-test was performed to compare quantitative parameters between the strabismic and non-strabismic eyes. Chi-square was performed to evaluate the significant differences in the prevalence of amblyopia, gender and eye laterality between the esotropic and exotropic groups. *P* < 0.05 was considered statistically significant.

## Results

The mean age at the time of the first surgery among all 1017 patients was 29.0 ± 12.4 years, ranging from 4 to 77 years. Of the total, 574 participants (56.4%) were males and 443 (43.6%) were females. Sensory exotropia was observed in 717 (70.5%) patients and sensory esotropia was found in 300 (29.5%) cases (*P*<.001). Also, 5 cases had sensory hypertropia without any horizontal deviation, which we did not compare with the two other groups because of the small number. There were 72 patients with a combination of both horizontal and vertical strabismus, including 57 with sensory esotropia and vertical deviation, and 15 with sensory exotropia and vertical deviation. Table 2 presents the number, mean, minimum and maximum angle of horizontal and vertical sensory strabismus at distance and near.

**Fig. 1:**
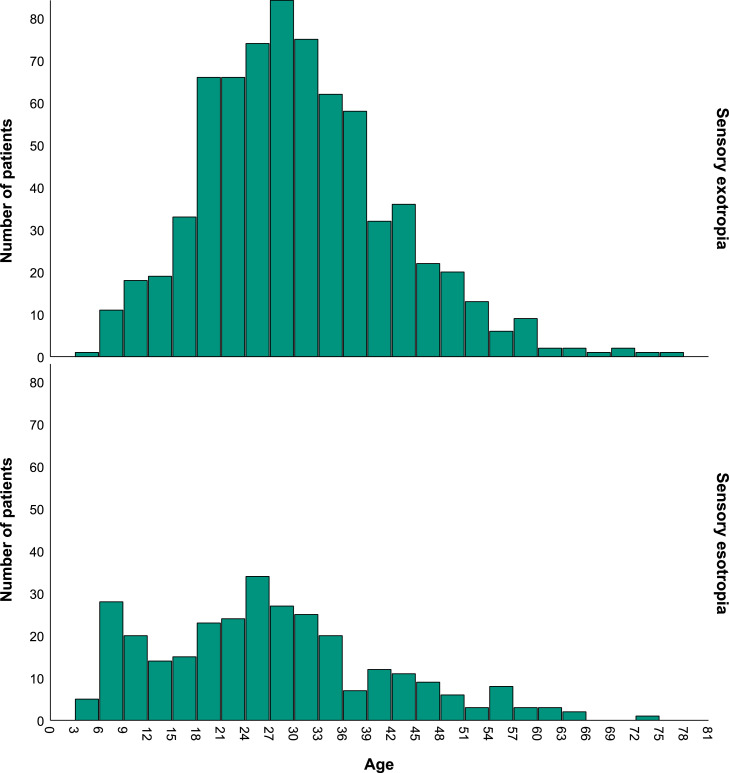
Age at the time of surgery of patients with sensory esotropia and exotropia.

There was a significant difference in age at the time of surgery between the esotropia and exotropia patients, such that sensory esotropia patients were operated on at a younger age (26.7 ± 14.1) than the sensory exotropia patients (30.1 ± 11.6) (*P*<.001). The age distributions of patients with sensory esotropia and sensory exotropia are reported in Fig. 1. As illustrated in this figure, the highest frequency age range of patients who underwent surgery in the sensory exotropia was in “27 to 30″ and then “24 to 27″ and “30 to 33″ years, whereas, in patients with sensory esotropia, it was “24 to 27″ and then “6 to 9″ years and “21 to 24″.

**Fig. 2:**
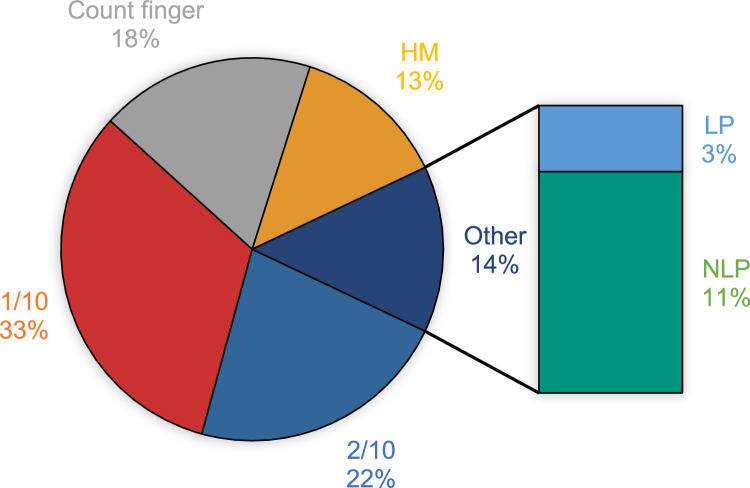
The percent frequency distribution of corrected distance visual acuity in the strabismic eye of patients with sensory strabismus. HM; hand motion, LP; light perception, NLP; no light perception.

Age at time of surgery, best corrected distance visual acuity, and refraction (sphere, cylinder, and spherical equivalent) in the strabismic and non-strabismic eyes in patients with sensory esotropia and sensory exotropia are reported in Table 1. The mean best corrected distance visual acuity in the strabismic and non-strabismic eyes was 1.40 ± 0.75 and 0.05 ± 0.13 logMAR (*P*<.001). Also, the best corrected distance visual acuity in the strabismic eyes, in the patients with sensory exotropia was significantly worse than the patients with sensory esotropia (*P*<.001). The most frequent best corrected distance visual acuity in the strabismic eye was 1/10 (1 logMAR) found in 330 (32.4%), and then 2/10 (0.7 logMAR) observed in 226 (22.2%) cases. The distribution of best corrected distance visual acuity in the strabismic eye of patients with sensory strabismus is shown in Fig. 2.

**Table 1:** Age, CDVA and refraction (sphere, cylinder and SE) in the strabismic and non-strabismic eyes in patients with sensory exotropia and sensory esotropia.

				Number	Minimum	Maximum	Mean ± SD	*P*-value
Age at first surgery (year)			Sensory XT	717	5.0	77.0	30.1 ± 11.6	<0.001
			Sensory ET	300	4.0	74.0	26.7 ± 14.1	
			Total	1017	4.0	77.0	29.1 ± 12.5	
CDVA (logMAR)	Strabismic eye		Sensory XT	717	0.70	2.90	1.46 ± 0.79	<0.001
			Sensory ET	300	0.70	2.90	1.25 ± 0.64	
			Total	1017	0.70	2.90	1.40 ± 0.75	
	Non-strabismic eye		Sensory XT	717	0.00	1.00	0.05 ± 0.12	.123
			Sensory ET	300	0.00	1.00	0.06 ± 0.15	
			Total	1017	0.00	1.00	0.05 ± 0.13	
Refraction (diopter)	Strabismic eye	Sphere	Sensory XT	717	−26.00	16.50	−0.01 ± 5.26	<0.001
			Sensory ET	300	−18.00	11.25	1.06 ± 3.51	
			Total	1017	−26.00	16.50	0.31 ± 4.83	
		Cylinder	Sensory XT	717	0.00	10.00	1.41 ± 1.77	.004
			Sensory ET	300	0.00	7.00	1.07 ± 1.36	
			Total	1017	0.00	10.00	1.31 ± 1.66	
		Spherical equivalent	Sensory XT	717	−29.00	16.50	−0.72 ± 5.42	<0.001
			Sensory ET	300	−19.00	11.25	0.53 ± 3.53	
			Total	1017	−29.00	16.50	−0.35 ± 4.97	
	Non-strabismic eye	Sphere	Sensory XT	717	−16.50	7.50	−0.13 ± 1.60	<0.001
			Sensory ET	300	−24.00	12.50	0.63 ± 2.53	
			Total	1017	−24.00	12.50	0.09 ± 1.96	
		Cylinder	Sensory XT	717	0.00	8.00	0.58 ± 0.88	.318
			Sensory ET	300	0.00	5.00	0.64 ± 0.89	
			Total	1017	0.00	8.00	0.59 ± 0.88	
		Spherical equivalent	Sensory XT	717	−17.75	6.50	−0.42 ± 1.76	<0.001
			Sensory ET	300	−24.00	12.50	0.31 ± 2.54	
			Total	1017	−24.00	12.50	−0.20 ± 2.05	

CDVA <20/400 was considered as follows: finger count: 2.0 logMAR; hand motion: 2.3 logMAR; light perception: 2.6 logMAR; and no light perception (NLP) = 2.9 logMAR.

CDVA: corrected distance visual acuity, SE: spherical equivalent, XT: exotropia, ET: esotropia.

Regarding the refractive errors, in both the strabismic and non-strabismic eyes, the mean sphere and spherical equivalent in patients with sensory esotropia was significantly more hyperopic than in cases with sensory exotropia (*P*<.001). The cylindrical component was significantly higher in patients with sensory exotropia than in patients with sensory esotropia only in the strabismic eye (*P=*.004). Analyzing the refractive errors in the non-strabismic eye showed that in 717 patients with sensory exotropia, 499 (69.6%) cases showed emmetropia, whereas, 71 (9.9%) and 147 (20.5%) cases showed hyperopia and myopia, respectively. In the 300 patients with sensory esotropia, emmetropia, hyperopia and myopia were observed in 159 (53%), 113 (37.7%) and 28 (9.3%) cases, respectively.

The horizontal and vertical angles of deviation in patients with sensory exotropia and esotropia are shown in Table 2. As reported in this table, in the patients with sensory esotropia, the mean horizontal deviation at far and near was significantly higher than the patients with sensory exotropia (both *P*<.001). However, the mean vertical deviation at far and near was not significantly different between the two study groups (*P*=.22 and *P*=.22, respectively).

**Table 2:** The horizontal and vertical angles of deviation in patients with sensory exotropia and sensory esotropia.

				Number	Minimum	Maximum	Mean ± SD	*P*-value
Angle of deviation in prism diopter (Primary position)	Near	Horizontal	Sensory XT	717	16	105	33.79 ± 13.975	<0.001
			Sensory ET	300	14	105	39.48 ± 18.941	
			Total	1017	14	105	35.47 ± 15.783	
		Vertical	Sensory XT	717	0	40	0.88 ± 3.615	.217
			Sensory ET	300	0	37	0.59 ± 2.90	
			Total	1017	0	40	0.80 ± 3.42	
	Far	Horizontal	Sensory XT	717	14	105	33.40 ± 13.575	<0.001
			Sensory ET	300	12	105	38.42 ± 18.964	
			Total	1017	14	105	34.76 ± 15.481	
		Vertical	Sensory XT	717	0	40	0.90 ± 3.639	.220
			Sensory ET	300	0	37	0.60 ± 2.96	
			Total	1017	0	40	0.81 ± 3.45	

XT: exotropia, ET: esotropia.

The prevalence of moderate and severe amblyopia among all patients with sensory strabismus was 274 (26.9%) and 727 (71.5%), respectively (*P*<.001). Also, strabismus was found in the right eye of 484 (47.6%) patients and the left eye of 533 (52.4%) cases (*P*=.22). The frequency of gender and laterality at different severities of amblyopia in patients with sensory esotropia and sensory exotropia are reported in Table 3.

**Table 3:** The frequency of gender, laterality, and different severities of amblyopia in patients with sensory exotropia and sensory esotropia.

			Sensory XT	Sensory ET	All cases
Gender	Male		416 (58.0%)	158 (52.7%)	574 (56.4%)
	Female *P*-value		301 (42.0%)	142 (47.3%)	443 (43.6%)
			<0.001	.970	.576
Strabismic eye	Right		358 (49.9%)	126 (42.0%)	484 (47.6%)
	Left *P*-value		359 (50.1%)	174 (58.0%)	533 (52.4%)
			.356	.006	.124
Amblyopia	Unilateral	Moderate	184 (25.6%)	90 (30.0%)	274 (26.9%)
		Severe *P*-value	522 (72.8%)	205 (68.3%)	727 (71.5%)
			<0.001	<0.001	<0.001
	Bilateral		11 (1.5%)	5 (1.7%)	16 (1.6%)
	Total		717 (100.0%)	300 (100.0%)	1017 (100.0%)

XT: exotropia, ET: esotropia.

In the sensory exotropia, the most common surgery was unilateral lateral rectus recession combined with a medial rectus resection in the strabismic eye found in 414 (57.7%) patients, followed by one lateral rectus recession observed in 197 (27.5%) and one medial rectus resection seen in 51 (7.1%) patients. In the sensory esotropia, the most common surgery was unilateral medial rectus recession combined with a lateral rectus resection in the strabismic eye found in 164 (54.7%) patients, followed by one medial rectus recession observed in 92 (30.7%) and one lateral rectus resection seen in 29 (9.3%) patients. 398 (39.1%) patients needed more than one surgery.

## Discussion

Not many studies have compared the clinical characteristics of patients with two types of sensory strabismus: sensory exotropia and sensory esotropia. In this retrospective study, a large sample of patients with sensory strabismus was reviewed, and the results are reported here: (1) the prevalence of sensory exotropia was about 2.5 times more than the sensory esotropia, (2) most of the patients with sensory esotropia had a younger age at the time of surgery than the patients with sensory exotropia, (3) spherical component and spherical equivalent measurements were more hyperopic in both the strabismic and non-strabismic eyes of patients with sensory esotropia than the patients with sensory exotropia, (4) angle of deviation was significantly higher in far and near in patients with sensory esotropia than the patients with sensory exotropia, (5) most of the patients in both groups suffered from severe amblyopia and (6) the chance of reoperation was about 40% in these patients. The obtained results are discussed in detail below.

Most of the patients in our study showed sensory exotropia (70.5%), which is in accordance with what other studies have found so far.^10^^,^^16^^,^^27^ Unfortunately, we did not have the data on age at the time of visual impairment; however, the results of other studies with this data confirm that age at the onset of visual impairment plays an important role in determining the direction of the strabismus. Studies have shown that when sensory deviation occurs under the age of 5 years old, there is either an equal possibility^5^ or less difference^15^ for developing sensory esotropia or exotropia; however, when sensory strabismus occurs at an older age, there is a more possibility of developing sensory exotropia than esotropia.^9^^,^^15^^,^^16^

In addition to the age at the time of impairment, several other factors can determine the direction of sensory strabismus, including refractive error in the fellow eye, degree of visual acuity, and anatomical factors; however, there is still no agreement for the mentioned parameters.^3^^,^^4^^,^^13^^,^^14^ In a retrospective study, Park et al investigated the factors that could determine the direction of sensory strabismus and to analyze the stability of the strabismus after surgical intervention in 98 patients with sensory esotropia and exotropia who had been observed for at least five years.^16^ They found that the prevalence of sensory esotropia and sensory exotropia was 20.4% and 79.6%, respectively, which was approximately in accordance with our findings. Moreover, they mentioned refractive error in the sound eye and age at the onset of visual loss determined the direction of strabismus, such that the proportion of esotropia significantly increased when the refraction of the sound eye became more hyperopic. We also observed that hyperopic refractive error in the sound eye was accompanied by esotropia, and the presence of myopia in the sound eye was with exotropia in the strabismic eye. On the contrary, Yoon et el.^9^ showed no relationship between the refractive error in the sound eye and the direction of strabismus in the other eye. However, our results is based on the largest sample examined in the literature.

The best corrected distance visual acuity in patients with sensory exotropia was worse than the sensory esotropia. In line with our findings, Choi et al.^6^ evaluated 71 patients with sensory strabismus and organic amblyopia, aged under 16 years old and reported that patients with severely impaired visual acuity showed higher frequency of sensory exotropia. They also reported that most patients (82%) had sensory exotropia as per our finding (70.5%). However, in contrast with our study design, they excluded patients with functional amblyopia and found that organic amblyopia patients tended to develop sensory exotropia in patients younger than 16 years old. This might also be the reason that the majority of the patients in our study also developed sensory exotropia, possibly because the only functional reason for sensory strabismus is high degrees of anisometropia which might not be as common as other all underlying organic factors including media opacities and retina and optic nerve issues. Possibly, three factors including age at the time of visual impairment, refractive error in the sound eye, and best corrected distance visual acuity were the influencing factors in developing higher frequencies of sensory exotropia than sensory esotropia in our study.

Patients with sensory esotropia underwent surgery at younger ages than the sensory exotropia. This finding might be related to the age group of 6–9 years in sensory esotropia group who showed a high frequency of operation in this group; whereas, in sensory exotropia group, the most three higher frequency age groups were between 24 and 27, 27–30 and 30–33 years.

Another finding in our study was that patients with sensory esotropia showed a significantly higher amount of deviation than the sensory exotropia (about 5–6 prism diopter); however, in terms of the clinical findings, it might not be significant. Min et al^8^ showed a relationship between the duration of visual impairment and the amount of sensory exotropia, such that if the visual impairment occurred for <5 years, the amount of deviation was less (about 9 prism diopter) than when it occurred for >5 years. Unfortunately, we did not have the data on duration of impairment, but there might be a direct relationship between the duration of impairment and the magnitude of the deviation, as sensory esotropia usually occurs at a younger age.

As expected, most of the patients suffered from severe amblyopia (71.5%). Severe amblyopia is considered as a poor prognosis factor in providing optimal sensory and motor results following the strabismus surgery.^28^ Moreover, the correlation between the visual acuity of the strabismic eye and the success of strabismus surgery is an important factor in predicting long-term alignment outcomes. When the eye that is affected by strabismus has better preoperative visual acuity, the chances of achieving and maintaining good ocular alignment post-surgery are typically higher .^17^^,^^29^ In our study, multiple surgeries were necessary in 39.1% of cases, a figure that may be linked to the substantial prevalence of severe amblyopia, which corresponds with the preoperative low visual acuity observed in the strabismic eye.

This study was the first study that compared various preoperative clinical features of patients with sensory exotropia and sensory esotropia who underwent strabismus surgery. In addition, it has the largest sample size among publications concerning sensory strabismus. However, the predominant limitation of the present study was retrospective nature of the data collected, which limited access to the full profile of patients with sensory strabismus, including age at the time of visual impairment and binocular status, including binocular fusion, abnormal retinal correspondence, suppression and stereopsis, which were missing in most cases. In addition, we reviewed the data of patients with sensory strabismus who underwent surgery only in one center. In order to record visual acuity, we only included patients older than 3.5 years old; therefore, the information regarding patients younger than this age was not collected. Another limitation was that we did not record post-operative data of patients who underwent surgical intervention to compare their clinical features before and after surgery. Future studies may follow the post-operative binocular status of patients with sensory exotropia and esotropia who underwent surgery, including the alignment status of the eyes, the presence or absence of diplopia following surgery, and the requirement for reoperation.

In conclusion, within our cohort of 1017 patients undergoing surgical intervention, sensory exotropia presented with a markedly higher prevalence, 2.5 times more common than sensory esotropia. Most patients with sensory ET were operated on at a younger age, had better corrected distance visual acuity, more hyperopic spherical and spherical equivalent, and higher angle of deviation compared with patients with sensory XT. Many of these cases were accompanied by severe amblyopia, correlating with preoperative low visual acuity. Notably, this association was reflected in a considerable reoperation rate. These findings underscore the pivotal role of visual acuity as a prognostic indicator to influence the success of surgical alignment in individuals with sensory strabismus.
